# The impact of combined endodontic-orthodontic treatment on the recovery of masticatory function in patients with crown-root fractures of permanent maxillary anterior teeth

**DOI:** 10.12669/pjms.42.1.12794

**Published:** 2026-01

**Authors:** Nana Zhang, Lei Mi, Na Jing

**Affiliations:** 1 Nana Zhang Department of Stomatology, Yulin First Hospital, Yulin, Shaanxi Province 719000, P.R. China; 2 Lei Mi Department of Stomatology, Yulin Traditional Chinese Medicine Hospital, Yulin, Shaanxi Province 719000, P.R. China; 3 Na Jing Department of Stomatology, Yulin First Hospital, Yulin, Shaanxi Province 719000, P.R. China

**Keywords:** Combined endodontic-orthodontic treatment, Crown-root fractures of permanent maxillary anterior teeth, Masticatory function

## Abstract

**Objective:**

To explore the influence of combined endodontic-orthodontic treatment on the recovery of masticatory function in patients with crown-root fractures of permanent maxillary anterior teeth.

**Methodology:**

This retrospective analysis included clinical data of 118 patients with crown-root fractures of permanent maxillary anterior teeth admitted to Yulin First hospital from March 2020 to March 2024. Among them, 56 patients who received conventional restoration treatment were set as the control group, and 62 patients who received combined endodontic-orthodontic treatment were set as the study group. The treatment effects, oral-related indicators before and after treatment [gingival index (GI), periodontal probing depth (PPD), tooth mobility (TM)], bite force, masticatory efficiency, and subjective satisfaction were analyzed.

**Results:**

The total efficacy of the study group (95.16%) was higher than that of the control group (83.93%) (P < 0.05). After treatment, the levels of GI, PPD, and TM in both groups decreased compared to pre-treatment, and were considerably lower in the study group (P < 0.05). The excellent-good rate of bite force in the study group (93.55%) was higher than that in the control group (80.36%) (P < 0.05). Similarly, combined endodontic-orthodontic treatment was associated with a higher excellent-good rate of masticatory efficiency (93.55%) than that of the conventional restoration (78.57%) (P < 0.05). The subjective satisfaction in the study group (95.16%) was higher than that in the control group (82.14%) (P < 0.05).

**Conclusion:**

The combined endodontic-orthodontic treatment for crown-root fractures of permanent maxillary anterior teeth can effectively improve the oral condition, enhance the bite force and masticatory efficiency, with higher treatment effects and subjective satisfaction. This approach may represent a promising treatment option for patients with crown-root fractures of permanent maxillary anterior teeth, pending further validation through prospective and multicenter studies.

## INTRODUCTION

Crown-root fracture of permanent maxillary anterior teeth is a common hard-tissue injury, especially in children and adolescents, and is caused mainly by external impacts, accidental falls, etc.^1^,^2^ These fractures not only affect the facial aesthetics of patients but also severely impair their masticatory function, may lead to loss of tooth structure stability, and an increased risk of pulp exposure and infection, further impacting oral health and quality of life.^3^

Currently, neither simple endodontic treatment nor orthodontic treatment of crown-root fracture of permanent maxillary anterior teeth can achieve the ideal restoration effect.^4^,^5^ Traditional endodontic treatment mainly focuses on controlling infection and preserving the affected tooth, often ignoring the influence of tooth position and occlusal relationship on the recovery of masticatory function; orthodontic treatment can adjust the tooth position and occlusal relationship, but cannot effectively deal with tooth tissue defects and pulp lesions.^6^,^7^ In recent years, with the development of oral medicine, the concept of combined endodontic-orthodontic treatment has gradually emerged. By integrating the advantages of the two treatment methods, this approach can repair tooth tissues while improving tooth arrangement and occlusal state, providing a new direction for treating patients with crown-root fractures of permanent maxillary anterior teeth.^8^-^10^

The clinical efficacy of combined endodontic-orthodontic treatment in restoring masticatory function in patients with crown-root fractures of maxillary anterior permanent teeth remains insufficiently explored. Although previous studies have reported the use of such combined therapy in managing complex crown-root fractures,^5^,^6^,^10^ most have emphasized outcomes related to tooth preservation, esthetics, or periodontal status,^3^,^8^,^9^ with limited attention to functional recovery. In particular, few investigations have evaluated masticatory efficiency as a primary endpoint. To address this gap, the present retrospective study quantitatively assessed both occlusal force and masticatory efficiency in patients undergoing combined treatment, aiming to generate functional evidence to support its clinical application and provide a practical reference for the management of anterior crown-root fractures.

## METHODOLOGY

A total of 118 patients with crown-root fractures of permanent anterior teeth, treated at Yulin First Hospital between March 2020 and March 2024, were retrospectively included in this study. Of these, 56 patients who received conventional restorative treatment were assigned to the control group, while 62 patients who underwent combined endodontic-orthodontic therapy were included in the study group. All clinical information—including diagnostic records, radiographic findings, treatment procedures, and follow-up documentation—was extracted from the hospital’s Electronic Medical Record (EMR) system. To ensure data accuracy and reduce the risk of bias, two trained independent researchers performed data extraction using a standardized abstraction form, and any discrepancies were resolved by a senior stomatologist with over ten years of clinical experience. Only patients with complete treatment and follow-up records were included, and no cases experienced dropout or loss to follow-up during the 6-12 months observation period.

### Ethical approval:

The study was approved by the Ethics Committee of Yulin First Hospital (No. 2025-015, Date: May 30, 2025).

### Inclusion criteria:

- The patients were diagnosed with the crown and root fracture of upper permanent teeth by clinical examination and had complete medical records.
- Age 8-18 years old, in the early or middle stage of permanent dentition.
- The root development of the affected tooth was ≥ 50%, as assessed on periapical or panoramic radiographs, with root length reaching at least half that of adjacent fully developed teeth and showing signs of partial or progressing apical closure.
- The periodontal tissue of the affected tooth was healthy, with no obvious periodontal pocket.
- Patients and their families agreed to participate in the study and signed the informed consent.
- No systemic disease affecting the treatment.
- No serious disorder of occlusal relationship, which can be adjusted by orthodontics.
- No recent interventions related to dental treatment.

### Exclusion criteria:

- Crown-root fracture with severe alveolar bone fracture or displacement.
- Severe tooth damage, defined as root fracture combined with more than 50% crown loss, or clinically diagnosed residual crown or root; presence of pulp necrosis or periapical abscess.
- Severe periodontal disease, defined as probing pocket depth (PPD) >6 mm or radiographic evidence of alveolar bone loss exceeding 50% of the root length.
- Severe malocclusion, requiring orthognathic surgery.
- Mental illness, unable to undergo therapy.
- Hypersensitivity to therapy-related materials (such as adhesives).
- Systemic diseases affecting bone metabolism (such as hyperthyroidism).
- Contraindications of orthodontic treatment (such as severe temporomandibular joint disorder).
- Poor compliance, unable to complete regular follow-ups.

### Treatment methods:

### Conventional restorative treatment (control group):

Under local anesthesia, the high-speed turbine with fissure drill was used to open the pulp; the infected pulp tissue was thoroughly removed using the step-back method for root canal preparation; 17% ethylenediaminetetraacetic acid (EDTA) solution and 5.25 % sodium hypochlorite solution were used to alternatively irrigate the root canal to ensure that the smear layer of the root canal wall was removed cleanly. The root canal was then disinfected with calcium hydroxide paste for 1-2 weeks. After the symptoms were relieved, the root canal was filled with hot gutta-percha using the vertical compression technique and AH Plus root canal sealer (Dentsply Sirona).

The X-ray confirmed the filling effect. After 1-2 weeks of root canal therapy, if the crown defect area was small, 3MZ350 nano resin was used for direct bonding repair, and the tooth shape was restored through acid etching, bonding, layered filling, light curing, etc. If the defect area was large, root canal treatment was done first, a fiber post was fixed in the root canal with a matching bonding system. The shape of the core was molded with resin, and the zirconia all-ceramic crown or metal porcelain crown was made for restoration. After the repair was completed, the early contact point was marked with occlusal paper, and the restoration was carefully adjusted to ensure that the affected tooth and the opposite tooth formed a uniform and stable occlusal contact relationship without occlusal trauma.

### Endodontic-orthodontic combined treatment (study group):

The pulp was treated similarly to the conventional treatment. After 1-2 weeks of root canal treatment, the orthodontic treatment was carried out with 0.022 inch × 0.028-inch straight wire appliance; 0.014-inch nickel-titanium round wire was used as an initial arch wire. A continuous light force in the range of 20–50g was applied through a chain-like elastic ligature to gradually reduce and reposition the displaced teeth. The specific force value was not applied uniformly but was tailored according to individual characteristics. For younger patients or those with developing periodontium, lighter forces (close to 20g) were preferred. In contrast, patients with stable periodontal support and more pronounced displacement received higher forces (up to 50g), all within the physiologically safe range. Force design and adjustments were performed by the same experienced orthodontic specialist to ensure safety and treatment consistency. During orthodontic treatment, follow-up visits were conducted once a month. The arch wire was replaced on schedule according to the teeth’s movement, allowing them to be gradually aligned and the occlusion relationship to be adjusted. The entire orthodontic treatment period typically lasted 6-12 months. After orthodontic treatment, the appliance was removed, and the teeth were restored. The choice of restoration methods was the same as that of the control group. According to the degree of tooth defect, either by resin direct restoration, fiber post and core combined with a full crown restoration, or other means. To prevent periodontal tissue damage caused by plaque accumulation, ultrasonic scaling and manual scaling were carried out every 1-2 months, and patients were instructed to use dental floss, interdental brushes, and other oral cleaning tools.

### Observation Indicators:

The following indicators were collected:

### Treatment Efficacy:

Markedly Effective: Masticatory function returns to normal; no tooth pain or looseness; occlusal relationship is stable and functional; the restoration remains intact and stable; radiographs show complete or near-complete resolution of periapical radiolucency; probing pocket depth is ≤ 3 mm.

### Effective

Masticatory function is noticeably improved; mild discomfort may occur occasionally without affecting chewing; tooth mobility is within Grade I; the restoration is stable; radiographs show a reduction in the periapical shadow; probing pocket depth is ≤ 4 mm.

### Ineffective

No improvement or worsening of masticatory function; significant pain or mobility (mobility > Grade I); restoration failure (detachment or fracture); radiographs show unchanged or enlarged periapical lesions; probing pocket depth is > 4 mm.

To complement these categorical assessments, functional parameters such as bite force and masticatory efficiency were quantitatively measured and used to validate treatment outcomes. “Markedly effective” and “Effective” cases were included in the total effective rate.

### Oral-related indicators before and after treatment:

All post-treatment measurements of GI, PPD, and TM were conducted within one week after the completion of final prosthetic restoration, ensuring that periodontal tissues had stabilized after orthodontic and restorative interventions.

### Gingival Index (GI):

A combination of visual inspection and blunt-tipped periodontal probe examination was used. Four sites (mesial, middle, distal on the buccal side and the lingual side) of each tooth were evaluated, and the score of the most severely affected site was recorded. The GI was assessed using the Löe & Silness index (1963),^11^ a validated and widely accepted scoring system for gingival inflammation. Assessment criteria: 0 points for healthy gingiva with no inflammation; one point for mild gingival inflammation without bleeding on probing; two points for moderate gingival inflammation with bleeding on probing; three points for severe gingival inflammation with spontaneous bleeding or ulceration. All GI, PPD, and TM assessments were performed by the same calibrated attending stomatologist with over five years of clinical experience in periodontal evaluation to ensure inter-rater consistency.

### Probing Pocket Depth (PPD):

Standardized probing techniques were used. A graduated periodontal probe was inserted vertically into the periodontal pocket at six sites (mesial, middle, distal on the buccal and lingual sides) of each tooth, and the maximum probing depth was recorded.

### ooth Mobility (TM)

Forceps were used to hold the incisal edge of anterior teeth or the occlusal surface of posterior teeth. The teeth were pushed in the buccolingual, mesiodistal, and vertical directions to determine the degree of mobility. Grade-I: Buccolingual mobility amplitude < 1mm; Grade-II: Buccolingual mobility amplitude of 1 - 2mm, or with mesiodistal mobility; Grade-III: Buccolingual mobility amplitude>2mm, or accompanied by vertical mobility and displacement.

### Biting force:

A high-precision biting force sensor (Shanghai Yuanxi Instrument Co., Ltd) was used to measure the maximum biting force of the affected side and both sides. During measurement, the patient sat in an upright position and occluded naturally until the cusps interlocked. The force was measured three times, and the average value was taken. Excellent: After treatment, the biting force of the affected side recovers to ≥ 80% of that of the healthy side, or increases by ≥ 30% compared to before treatment. Good: The biting force of the affected side reaches 60%-79 % of that of the healthy side, or increases by 10%-29 %. Poor: The biting force of the affected side is < 60% of that of the healthy side, or increases by < 10% or even decreases. The excellent-good rate = (number of excellent cases + number of good cases) / total number of cases × 100%.

*Masticatory efficiency*, measured by the absorbance method. The patient was instructed to chew a standard test food (such as 5g of roasted peanuts) 20 times. The chewing residues were collected, dissolved in distilled water, and centrifuged to collect the supernatant. The absorbance value (A) was measured at a wavelength of 540nm using a spectrophotometer. The higher absorbance indicated finer residues and higher masticatory efficiency. Excellent: After treatment, the absorbance value increases by ≥ 25% compared to before treatment. Good: The absorbance value increases by 10% - 24%. Poor: The absorbance value increases by < 10% or decreases. The excellent-good rate = (number of excellent cases + number of good cases) / total number of cases × 100%. ....

### Subjective satisfaction:

Visual Analogue Scale (VAS, 0 - 10 points) was used to evaluate patients’ satisfaction with masticatory function, aesthetic effect, and comfort. A score of ≥ 8 points indicates satisfaction, 6 - 7 points indicates basic satisfaction, and < 6 points indicates dissatisfaction. The total satisfaction rate = (number of satisfied cases + number of basically satisfied cases) / total number of cases × 100%.

### Statistical analysis:

Data were analyzed using SPSS 25.0. For measurement data, Bartlett’s test of homogeneity of variances and Kolmogorov-Smirnov normality test were performed. When both tests confirmed homogeneity of variances and approximate normal distribution, the data were expressed as (Mean ± SD). An Independent-samples t-test was used for inter-group comparison, and a paired t-test was used for intra-group comparison. Enumeration data were expressed as [cases (%)], and χ² test was applied. A P-value < 0.05 indicated a statistically significant difference.

## RESULTS

There was no significant difference in gender, age, interval between fracture and admission, types of teeth, causes of injury, and other general data between the two groups (*P*>0.05; Table-I). The total efficacy of the study group (95.16%) was higher than that of the control group (83.93%) (P < 0.05; Table-II).

**Table-I:** General characteristics.

Items	Study group (n = 62)	Control group (n = 56)	t/χ² value	P - value
***Gender [n (%)]***				
- Male	36(58.06)	30(53.57)	0.241	0.623
- Female	26(41.94)	26(46.43)		
Age (Mean ± SD, years)	9.08±2.47	8.97±2.07	0261	0.795
Interval from fracture to admission (Mean ± SD, d)	2.49±1.05	2.64±1.17	0.734	0.464
***Type of affected tooth [n (%)]***				
- Central incisor	48(77.42)	41(73.21)	0.281	0.596
- Lateral incisor	14(22.58)	15(26.79)		
***Cause of injury [n (%)]***				
- Fall injury	33(53.23)	29(51.79)	0.670	0.715
- Traffic accident	21(33.87)	22(39.29)		
- Others	8(12.90)	5(8.93)		

**Table-II:** Treatment effect [n (%)].

Group	Number of cases	Markedly effective	Effective	Ineffective	Total effective rate
Study group	62	32(51.61)	27(43.55)	3(4.84)	59(95.16)
Control group	56	24(42.86)	23(41.07)	9(16.07)	47(83.93)
*χ²- value*					4.064
*P - value*					0.044*

* indicates P < 0.05.

Pre-treatment levels of GI, PPD, and TM were comparable in the two groups (P > 0.05). Table-III. After treatment, the levels of GI, PPD, and TM in both groups decreased and were significantly lower in the study group compared to the control group (P < 0.05). The excellent-good rate of occlusal force in the study group (93.55%) was higher than that of the control group (80.36%) (P < 0.05; Table-IV). The excellent-good rate of masticatory efficiency in the study group (93.55%) was superior to the control group (78.57%) (P < 0.05). Table-V and Table-VI demonstrates that the combined treatment approach was associated with considerably higher satisfaction than the conventional one (95.16% vs 82.14%, respectively; P < 0.05).

**Table-III:** Oral-related indicators ( Mean ± SD).

Time	Group	Number of cases	GI (points)	PPD(mm)	TM(grade)
Before treatment	Study group	62	2.12±0.41	3.13±0.62	1.52±0.37
	Control group	56	2.09±0.46	3.07±0.67	1.49±0.40
	*t - value*		0.375	0.505	0.423
	*P - value*		0.709	0.614	0.673
After treatment	Study group	62	1.14±0.29	1.51±0.25	0.49±0.18
	Control group	56	1.46±0.32	1.79±0.34	0.61±0.21
	*t - value*		5.699	5.129	3.341
	*P - value*		＜0.001*	＜0.001*	0.001*

GI: gingival index; PPD: probing pocket depth; TM: tooth mobility.

* indicates P < 0.05.

**Table-IV:** Occlusal force [n (%)].

Group	Number of cases	Excellent	Good	Poor	Excellent-good rate
Study group	62	31(50.00)	27(43.55)	4(6.45)	58(93.55)
Control group	56	23(41.07)	22(39.29)	11(19.64)	45(80.36)
*χ²- value*					4.614
*P - value*					0.032*

* indicates P < 0.05.

**Table-V:** Masticatory Efficiency [n (%)].

Group	Number of Cases	Excellent	Good	Poor	Excellent-good rate
Study group	62	30(48.39)	28(45.16)	4(6.45)	58(93.55)
Control Group	56	21(37.50)	23(41.07)	12(21.43)	44(78.57)
*χ²- value*					5.631
*P - value*					0.018*

* indicates P < 0.05.

**Table-VI:** Subjective Satisfaction [n (%)].

Group	Number of Cases	Satisfied	Basically satisfied	Dissatisfied	Total satisfaction rate
Study group	62	36(58.06)	23(37.10)	3(4.84)	59(95.16)
Control Group	56	31(55.36)	15(26.79)	10(17.86)	46(82.14)
*χ²- value*					5.087
*P - value*					0.024*

* indicates P < 0.05.

## DISCUSSION

This study systematically explored the impact of combined endodontic-orthodontic treatment on the restoration of masticatory function in patients with crown-root fractures of maxillary anterior permanent teeth. The results showed that combined endodontic-orthodontic treatment was significantly superior to conventional restoration treatment in terms of improving treatment effects, oral health indicators, and masticatory function. This finding provides an important basis for optimizing clinical treatment strategies. Treating crown-root fractures of maxillary anterior permanent teeth is complex and needs to take into account dental restoration, occlusal reconstruction, and periodontal health maintenance. Therefore, traditional single-treatment methods often cannot achieve ideal results.^12^ The results of this study showed that the excellent-good rates of occlusal force (93.55%) and masticatory efficiency (93.55%) in the study group were significantly higher than those in the control group.

This result reveals the unique advantages of combined endodontic-orthodontic treatment in promoting the restoration of masticatory function in patients with this type of fracture. From a biomechanical perspective, the destruction of the dental support structure after a crown-root fracture of a maxillary anterior permanent tooth leads to abnormal stress distribution. Simple restoration only focuses on the morphological restoration of the tooth without correcting the decrease in masticatory efficiency caused by tooth displacement or occlusal disorders.^11^ Orthodontic treatment can reposition the affected tooth through gentle traction and re-establish physiological occlusal contact points, so that the occlusal force is evenly distributed on the periodontal ligament, reducing the damage to the periodontal tissue caused by local stress concentration.^13^ At the same time, the physiological movement of the tooth root during orthodontic treatment can stimulate the proliferation of periodontal ligament cells and alveolar bone remodeling, enhancing the strength of periodontal supporting tissues and providing a mechanical basis for the restoration of masticatory function.^13^ In addition, endodontic treatment effectively controls infection and restores the integrity of hard dental tissues. It forms a dual optimization of function and structure with orthodontic treatment, ultimately significantly improving masticatory efficiency and occlusal force.^13^

This study also found that the combined approach was associated with a considerable decrease in GI, PPD, and TM compared to the conventional treatment of the control group. The result indicates that combined endodontic-orthodontic treatment has significant advantages in improving periodontal health in patients with crown-root fractures of maxillary anterior permanent teeth. Such fractures are often accompanied by periodontal tissue damage and plaque retention, which, if not intervened in time, may trigger periodontal inflammation, further destroying the tooth-supporting structures.^14^ Aligning teeth and improving the adjacent relationship during orthodontic treatment can reduce food impaction, plaque adhesion, and the risk of periodontitis. Moreover, the continuous effect of orthodontic force can promote alveolar bone remodeling and enhance the attachment stability of periodontal ligament fibers, thereby reducing tooth mobility.^15^

Furthermore, adjusting the tooth position through orthodontics before tooth restoration can improve the marginal fit of the restoration, thereby avoiding periodontal irritation caused by overhanging restorations or poor adaptation.^16^ A combined endodontic-orthodontic approach includes root canal treatment, thereby completely removing infected substances within the root canal and eliminating the irritation of inflammation to the pulp and periapical tissues. This allows avoiding the limitation of masticatory function on the affected side due to pain or inflammation. Moreover, restoration of hard dental tissues can restore the shape and strength of the tooth, thereby enhancing its ability to withstand forces during mastication. Such dual optimization of function and structure with orthodontic treatment ultimately significantly improves masticatory efficiency and occlusal force.^17^ The significant improvement of periodontal indices in this study confirms the synergistic effect of combined endodontic-orthodontic treatment in preventing periodontal complications and promoting periodontal tissue repair. It is worth noting that the assessment of masticatory efficiency using the absorbance method may be influenced by individual differences in chewing patterns and laterality.^18^ To mitigate inter-subject variability, we standardized the procedure by instructing all patients to chew a fixed amount (5 grams) of roasted peanuts exactly 20 times using the affected side, under direct supervision by trained clinicians. All chewing residues were processed in a consistent laboratory setting and measured by the same calibrated examiner using a spectrophotometer. While this method has been widely validated in prior studies,^19^ we additionally confirmed repeatability in a subset of patients through duplicate testing, which showed high intra-sample consistency. Nevertheless, we acknowledge that this method lacks multidimensional feedback on mastication and suggest that future studies incorporate artificial test foods with known fragmentation profiles or use mechanical masticatory simulators to enhance objectivity and reproducibility.

This study found that the total efficacy of treatment in the study group (95.16%) was significantly higher than that in the control group (83.93%) (P < 0.05). This result reflects the superiority of combined treatment in terms of overall treatment efficacy. A conventional restoration treatment focuses solely on dental defects and endodontic lesions, overlooking the impact of abnormal tooth position on the treatment outcome, which may lead to issues such as shortened restoration lifespan and occlusal trauma. In contrast, combined treatment pre-adjusts the position of the affected tooth during the orthodontic stage, making subsequent dental restoration more in line with physiological morphology and mechanical requirements, and reducing the risk of failure due to unreasonable restoration design.^20^ In addition, orthodontic treatment can enhance the aesthetics of the anterior teeth and improve occlusal function in patients, thereby increasing their self-confidence and treatment compliance, and indirectly promoting the improvement of treatment outcomes.^21^ The step-by-step implementation of combined treatment (first endodontic treatment, then orthodontics, and finally restoration) helps to reconstruct function and aesthetics by controlling infection, avoiding the mutual interference of multidisciplinary treatments, and thereby further increasing the treatment success rate.

This study demonstrated that patients treated by the combined approach reported considerably superior subjective satisfaction (95.16%) compared to patients treated by the conventional method (82.14%). This result further confirms the advantages of combined endodontic-orthodontic treatment in meeting patients’ functional and aesthetic needs. As a key area in smile aesthetics, the damage to maxillary anterior permanent teeth not only affects masticatory function but also adversely impacts patients’ mental health. Combined endodontic-orthodontic treatment enhances tooth alignment and occlusal relationship through orthodontic intervention. It restores the shape and color of the tooth through dental restoration, resulting in a dual improvement in both function and aesthetics. In addition, the significant improvement in masticatory function directly improves diet quality and life experience of patients, increasing their acceptance of the treatment. The improvement in subjective satisfaction in this study also suggests that clinical treatment should pay more attention to the multi-dimensional needs of patients, combining functional restoration with aesthetic repair to enhance the overall value of treatment.

This study possesses several notable strengths. First, a standardized treatment protocol was applied across all patients, reducing variability and ensuring procedural consistency. Second, the use of both objective and subjective outcome measures—including periodontal indicators (GI, PPD, TM), quantitative bite force, masticatory efficiency via absorbance method, and patient-reported satisfaction—allowed for a comprehensive assessment of therapeutic outcomes. Third, the study utilized real-world clinical data collected from a well-defined patient population within a single institution, enhancing the clinical applicability of the findings. Lastly, the inclusion of a control group and appropriate statistical analysis strengthened the internal validity of the results.

Despite these strengths, further research is warranted to build upon our findings. Future studies should adopt prospective and multicenter designs to increase generalizability and reduce selection bias. Long-term follow-up is necessary to assess the durability of treatment outcomes and the incidence of late complications. Additionally, incorporating standardized imaging-based indices, such as the Periapical Index, would enhance the objectivity of radiographic evaluations. It is also recommended to include validated oral health-related quality-of-life (OHRQoL) instruments, such as OHIP-14 or GOHAI, to capture patient-centered outcomes more effectively. These refinements would contribute to a more robust and holistic framework for evaluating the clinical value of combined endodontic-orthodontic treatment.

### Limitations

First, as a retrospective single-center study, it may be subject to inherent selection bias and unmeasured confounding factors, which could affect the internal validity and generalizability of the results. Second, no long-term follow-up was performed, making it impossible to assess the durability of therapeutic outcomes or the incidence of late complications. Third, the study did not stratify patients based on different types of crown-root fractures (e.g., horizontal vs. oblique), which may influence treatment planning and prognosis. Future prospective randomized controlled trials should explore these fracture subtypes in combination with biomechanical analysis and individualized treatment planning, including personalized orthodontic force levels and timing of restorative interventions. Additionally, due to the retrospective design, digital occlusal models, bite registration records, and T-Scan data were not available. Although bite force measurements were used to estimate occlusal recovery, they may not fully capture the dynamic characteristics of occlusal function. Future prospective studies should incorporate digital occlusal analysis tools for more comprehensive and objective assessment of occlusal reconstruction. Furthermore, patient satisfaction was assessed using a simple visual analog scale (VAS) with three outcome categories, which lacks the multidimensionality of validated oral health-related quality-of-life (OHRQoL) instruments. Consequently, the psychological, functional, and social aspects of treatment impact could not be fully captured. The use of validated questionnaires such as OHIP-14 or GOHAI in future research is recommended to better reflect patient-reported outcomes. Finally, although treatment efficacy was judged using a combination of clinical signs, radiographic findings, and probing depth, certain components—such as pain perception and restoration stability—remain subjective. To improve objectivity and reproducibility, future studies should adopt standardized radiographic indices (e.g., the Periapical Index) and develop multidimensional, quantitative evaluation frameworks that integrate imaging, clinical function, and patient-centered outcomes. Furthermore, although treatment efficacy in this study was categorized using a combination of clinical signs, radiographic changes, and periodontal probing depth, we acknowledge that certain components, such as pain perception and restoration stability, retain subjective elements. To enhance reproducibility and objectivity, future studies should consider adopting standardized radiographic indices (e.g., Periapical Index) and defining quantitative radiographic thresholds. Moreover, the integration of multidimensional, quantitative assessment models combining imaging, clinical function, and patient-reported outcomes may provide a more comprehensive framework for evaluating treatment success.

## CONCLUSION

Due to the collaborative effect of multiple disciplines, combined endodontic-orthodontic treatment is significantly superior to conventional restoration treatment in improving the masticatory function of patients with crown-root fractures of maxillary anterior permanent teeth. The combined approach is associated with superior maintenance of periodontal health and enhanced clinical efficacy. This treatment model not only solves the dual problems of dental restoration and occlusal reconstruction but also considers patients’ functional needs and aesthetic expectations. Further mechanistic studies with long-term follow-up are needed to provide a more sufficient theoretical basis and clinical evidence for the standardized application of the combined endodontic-orthodontic treatment in this group of patients.

### Authors’ contributions:

**NZ:** Literature search, study design and manuscript writing.

**NZ, LM and NJ:** Data collection, data analysis and interpretation. Critical Review.

**NZ:** Manuscript revision and validation and is responsible for the integrity of the study.

All authors have read and approved the final manuscript.
